# The complete mitochondrial genome of *Oenopia sauzeti* (Coleoptera: Coccinellidae)

**DOI:** 10.1080/23802359.2021.1972859

**Published:** 2021-09-13

**Authors:** Zhi-yan Wei, Ren-huai Dai

**Affiliations:** Institute of Entomology, The Provincial Key Laboratory for Agricultural Pest Management of Mountainous Region, Guizhou University, Guiyang, Guizhou, PR China

**Keywords:** Mitogenome, lady beetles, *O. sauzeti*, phylogeny

## Abstract

*Oenopia sauzeti* Mulsant are predators of various aphids, such as *Myzus persicae*, *Rhopalosiphum maidis*, and *Sitobion avenae*. We sequenced the mitochondrial genome (mitogenome) of *O. sauzeti*. Consistent with other mitogenomes of Coleoptera, the circular mitogenome of *O. sauzeti* consists of 17,630 bp, including 13 PCG genes, 2 rRNA genes, and 22 tRNA genes. The mitogenome differs in 2 long non-coding regions of 1285 bp and 1843 bp. The 22 tRNA genes products are folded into the typical cloverleaf secondary structure, except *trnS1*, which lacks the dihydrouridine (DHU) arm. The base composition is AT-biased (80.1%). The phylogenetic tree confirms monophyly at the genus level within the Coccinellinae and supports *O. sauzeti* as a sister group to *Aiolocaria hexaspilota*.

Lady beetles (e.g. *Oenopia sauzeti* Mulsant, Coccinellidae), belonging to the Coleoptera, are a diverse group with more than 6000 species worldwide (Che et al. 2021). About 680 species are recorded from China (Pang 2002). *Oenopia sauzeti* are predators of various aphids, such as *Myzus persicae*, *Rhopalosiphum maidis*, and *Sitobion avenae*. In this study, we sequenced and characterized for the first time the complete mitogenome of *O. sauzeti* using next-generation sequencing. This work will help elucidate the relationship between the mitogenomes and the phylogeny of the species. Sequences obtained in this study provide a reference for further studies on the genetic characterization of *O. sauzeti*.

Total genome DNA was extracted from male adult beetles collected in Jinzhu Town, Guiyang, China (106°66′20′′E, 26°50′13′′N) in July 2020. A DNA sample was deposited at the Institute of Entomology, Guizhou University, Guiyang, China (Ren-huai Dai, dmolbio@126.com) under the voucher number GUGC-CO-0102. Sequencing used a Illumina HiSeq 4,000 (Berry Genomic, Beijing, China; 6GB raw data). Clean sequences were assembled using Geneious Primer (v. 2020.0.5) with *Harmonia axyridis* (GenBank: KR108208) as the reference (Seo et al. 2019). Assembled mitogenome sequences were annotated using the MITOS web server with the invertebrate genetic code (Bernt et al. 2013). Additional verification of transfer RNA (tRNA) used tRNAscan-SE (Schattner et al. 2005).

*Oenopia sauzeti* mitogenome length is 17,630 bp (GenBank accession number MW530420) and includes 13 protein-coding genes (PCGs), 2 ribosomal RNA genes (rRNAs), and 22 transfer RNA genes (tRNAs). Nucleotide composition is AT-biased (A: 41.3%, C: 11.6%, G: 8.3%, T: 38.8%). All PCGs start with an ATD (ATA, ATT, ATG) codon. Seven, three and three PCGs stop with the termination codons TAA, TAG, and single T, respectively. The size of tRNAs ranges from 56 bp (*trn-S1*) to 70 bp (*trn-K*). The mitogenome also displays 2 long non-coding regions like regions found in *A*. *hexaspilota* (MK583344) and *Cheilomenes sexmaculata* (KM244706). The first region (CN1, 1853 bp) is located between 12S rRNA and *trn-I*, and the second is located between *trn-I* and *trn-Q* (1285 bp).

Phylogenetic analyses used 17 complete or near-complete mitogenome sequences, 15 from the Coccinellinae subfamily, and one from each outgroup – Epilachninae and Molytinae. Phylogenetic analysis employed alignments of 13 PCG datasets and all codon positions within its 10,839 bp. Each PCG sequence was aligned using the MAFFT algorithm in TranslatorX. Relationships were reconstructed with IQ-TREE using an ultrafast bootstrap approximation with 10,000 replicates and concatenated datasets. BI used MrBayes 3.2.6 . Two independent runs with four simultaneous Markov chains (one cold and three incrementally heated at *T* = 0.2) were performed for 100 million generations, with sampling every 1000 generations. Monophyly at the genus level within the subfamily is strongly supported in the phylogenetic trees. *Oenopia sauzeti* as a sister group to *A*. *hexaspilota* is also well supported (PP > 0.94, BS = 100) (Figure 1). However, the Coccinellidae consists of over 6000 species in 360 genera, only a few genera have published mitogenomes, therefore, we hope our data can be useful for understanding the phylogeny and evolution of Coccinellidae.

**Figure 1.**
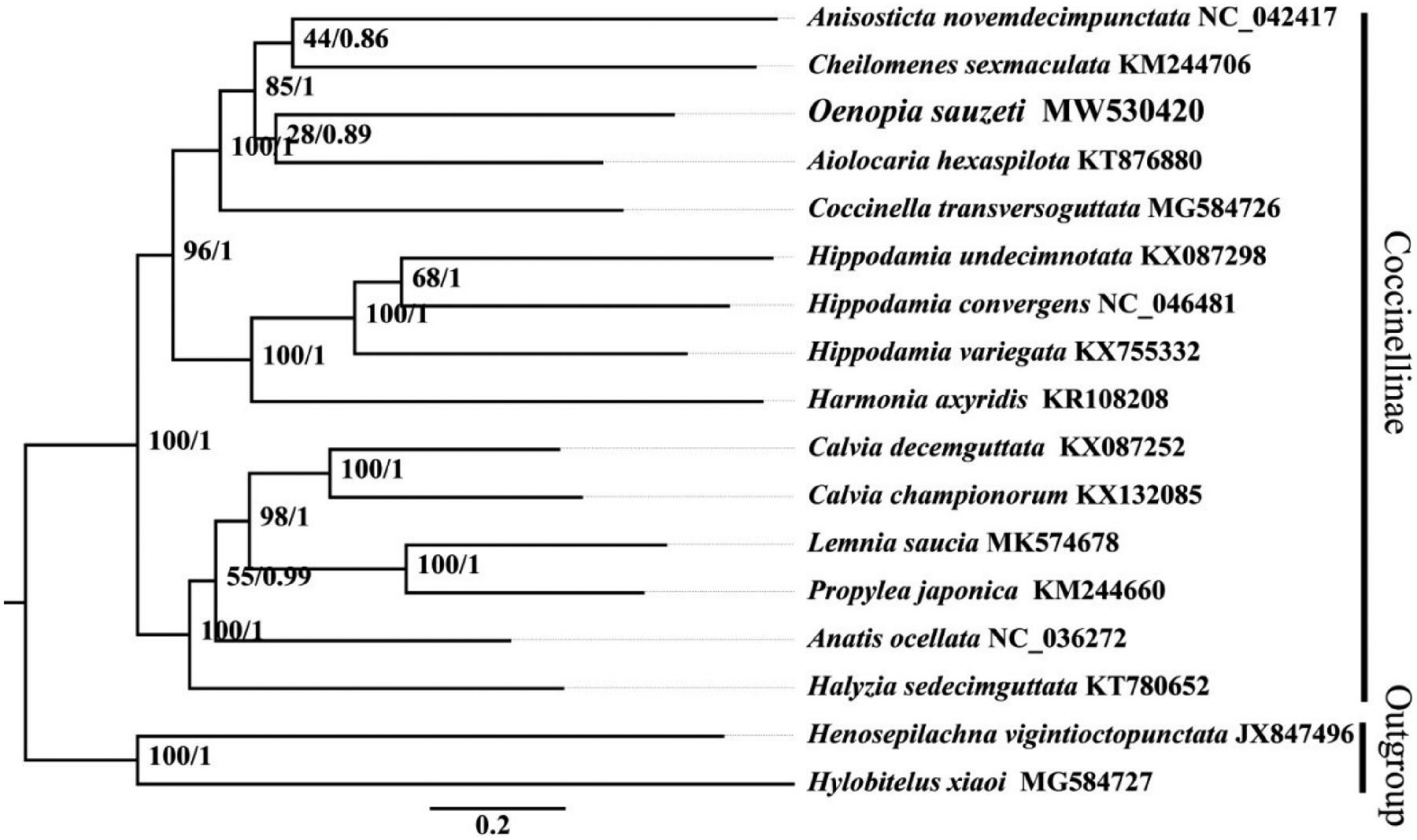
Phylogenetic analyses of the Coccinellinae based on concatenated codon positions of 13 PCGs. Analyses used IQ-TREE and MrBayes 3.2.6 software. Numbers at nodes are bootstrap values. Accession numbers for each species was indicated after scientific names.

## Data Availability

Sequence data from this study are available in GenBank of the NCBI at https://www.ncbi.nlm.nih.gov/ under accession no. MW530420. Associated BioProject, SRA, and Bio-Sample numbers are PRJNA698471, SRR13594807, and SAMN17720479, respectively.”
